# Experience of a multidisciplinary task force with exome sequencing for Mendelian disorders

**DOI:** 10.1186/s40246-016-0080-4

**Published:** 2016-06-28

**Authors:** S. Fokstuen, P. Makrythanasis, E. Hammar, M. Guipponi, E. Ranza, K. Varvagiannis, F. A. Santoni, M. Albarca-Aguilera, M. E. Poleggi, F. Couchepin, C. Brockmann, A. Mauron, S. A. Hurst, C. Moret, C. Gehrig, A. Vannier, J. Bevillard, T. Araud, S. Gimelli, E. Stathaki, A. Paoloni-Giacobino, A. Bottani, F. Sloan-Béna, L. D’Amato Sizonenko, M. Mostafavi, H. Hamamy, T. Nouspikel, J. L. Blouin, S. E. Antonarakis

**Affiliations:** Service of Genetic Medicine, University Hospitals of Geneva, Geneva, Switzerland; Department of Genetic Medicine and Development, University of Geneva, 1 rue Michel-Servet, 1211 Geneva, Switzerland; iGE3, Institute of Genetics and Genomics of Geneva, Geneva, Switzerland; Institute for Ethics, History, and the Humanities, Geneva University Medical School, Geneva, Switzerland

## Abstract

**Background:**

In order to optimally integrate the use of high-throughput sequencing (HTS) as a tool in clinical diagnostics of likely monogenic disorders, we have created a multidisciplinary “Genome Clinic Task Force” at the University Hospitals of Geneva, which is composed of clinical and molecular geneticists, bioinformaticians, technicians, bioethicists, and a coordinator.

**Methods and results:**

We have implemented whole exome sequencing (WES) with subsequent targeted bioinformatics analysis of gene lists for specific disorders. Clinical cases of heterogeneous Mendelian disorders that could potentially benefit from HTS are presented and discussed during the sessions of the task force. Debate concerning the interpretation of identified variants and the content of the final report constitutes a major part of the task force’s work. Furthermore, issues related to bioethics, genetic counseling, quality control, and reimbursement are also addressed.

**Conclusions:**

This multidisciplinary task force has enabled us to create a platform for regular exchanges between all involved experts in order to deal with the multiple complex issues related to HTS in clinical practice and to continuously improve the diagnostic use of HTS. In addition, this task force was instrumental to formally approve the reimbursement of HTS for molecular diagnosis of Mendelian disorders in Switzerland.

## Background

**Fig. 1:**
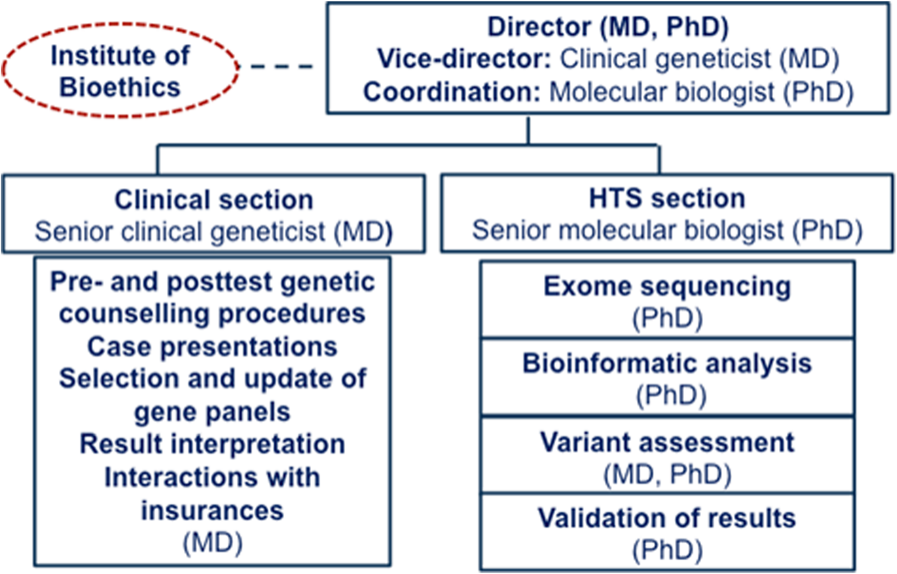
Organization chart of the Genome Clinic Task Force

Since the technological and bioinformatics developments of high-throughput sequencing (HTS) and the use of exome sequencing for the discovery of new genes causative of Mendelian disorders [1, 2], this technology has been rapidly and widely integrated in the clinical setting [3] as it outperforms previously used methods in diagnostic yield, time, and cost-effectiveness [4]. However, the use of HTS technology in the clinical setting brings its own set of challenges (7), although many of them were already encountered during the introduction of other genomic diagnostic methods such as array CGH. The main challenges of diagnostic HTS include pre- and post-HTS counseling with appropriate and adapted informed consent [5, 6], bioinformatics analysis setup and validation [7], variant interpretation and classification [8–10], specific policies concerning the identification and disclosure of variants not directly linked to the patient’s phenotype [11], validation of HTS as a diagnostic test that conforms to quality control standards [12], data storage and accessibility, and reimbursement issues [13], as well as updates and follow-up strategies. In order to optimally integrate HTS into the clinical practice and to continuously improve this novel and rapidly evolving diagnostic approach, we have realized quite early in the process the need for a multidisciplinary approach. Accordingly, the Genome Clinic Task Force (GCTF) was established in 2012, with the specific objective to provide a platform for regular exchanges of all involved specialists in order to find solutions for the various types of problems and concerns that we may encounter by performing HTS in our clinic. Currently, this task force meets once per week and is composed of roughly 25 specialists and a coordinator, including clinical geneticists (consultants and trainees), molecular biologists, scientists, bioinformaticians, bioethicists, and technicians (Fig. 1).

In this review, we present the composition, practices, and workflow of the GCTF, the results obtained to date, the challenges we have encountered, the reimbursement directives that were officially introduced in Switzerland in January 2015 by the Swiss Federal Office of Public Health (SFOPH), and the lessons learned from this experience.

## The Genome Clinic Task Force (GCTF) of the University Hospitals of Geneva

Figure 1 shows the organization of the GCTF working group as well as the tasks that the two sections (clinical and laboratory) have to fulfill. The head of our Genetics Institute, an MD, PhD, is the director of the task force. The coordinator is a trained PhD molecular biologist with experience in health policy and diagnostic issues. The role of the coordinator is to perform the preparatory work of each GCTF session, to formalize the procedures, to record the minutes of all GCTF sessions, and to handle relevant administrative tasks. The clinical section consists of the clinical geneticists of our service, who present patients to the task force and critically examine the indications of HTS for each patient, as well as providing their input regarding the clinical interpretation of identified variants. The HTS laboratory section is headed by a senior molecular biologist with appropriate qualifications for molecular diagnostic services and subdivided in a sequencing, bioinformatics, and analysis groups. Finally, two bioethicists from the Institute of Bioethics of the University of Geneva are participating in the weekly meetings. Their participation helps to immediately address ethical issues that may arise during the discussions. The profession of the genetic counselor (as it is defined in the USA) is not formally recognized as such in Switzerland, and thus genetic counselors are not included in the task force.

### Standard operating procedure

**Fig. 2:**
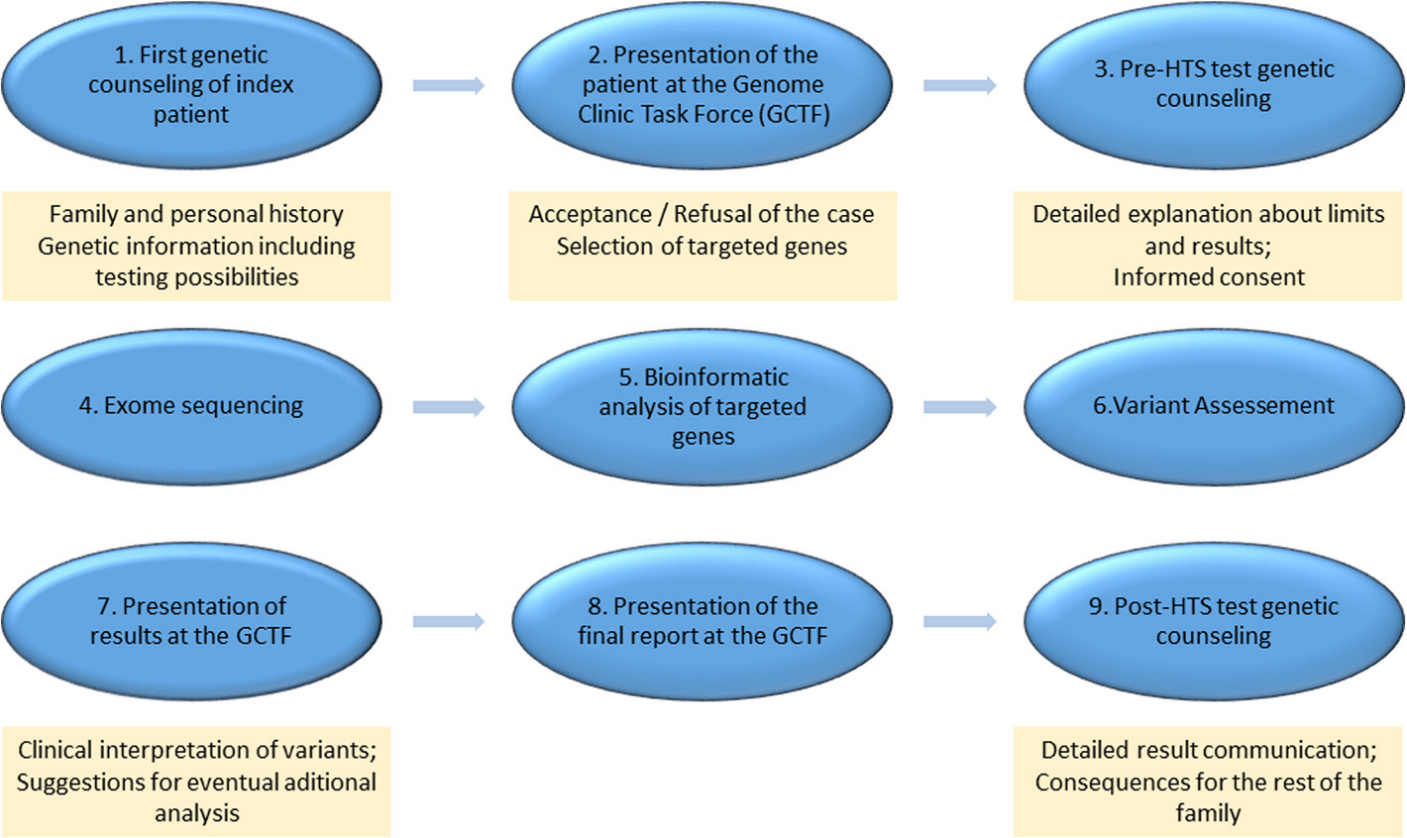
Overview of the practical steps (1–9) of the Genome Clinic Task Force

The different steps of the diagnostic workflow are shown in Fig. 2 and illustrated by an example. This standard operating procedure was among the initial objectives of the GCTF and is regularly reviewed according to the evolution of this diagnostic field. As minutes of all GCTF meetings are written, all discussions and decisions taken can be referred to and reevaluated according to new experiences, international recommendations, and practical considerations. As shown in the flowchart, every case that undergoes diagnostic HTS is discussed at least three times in the GCTF: a first time before the test is performed (step 2), a second time during the preliminary report (step 7), and a third time (step 8) during the presentation and debate of the final report. The following paragraphs provide more details on the operating procedure.

### Pre-test considerations (steps 1, 2, and 3 of Fig. 2)

Clinical cases that have been seen for genetic counseling at the Genome Clinic (step 1) and who may benefit from HTS are presented by one of the clinical geneticists at the GCTF (step 2) in order to evaluate within the task force the clinical and reimbursement aspects of the case and to decide whether the patient is appropriate for a HTS approach. In general, we accept patients who suffer from a likely heterogeneous Mendelian disorder with at least one known clearly pathogenic gene or patients with a developmental delay of unknown origin. The cost of the HTS analysis has to be less than that of Sanger sequencing for the corresponding genes. Physicians from other medical specialties may also present their cases during the sessions. These presentations include a detailed family history, personal medical history, photos if available, genetic tests that have already been performed, and the list of genes proposed to be tested. So far, 246 patients have been presented to the GCTF and 240 cases were accepted. Two were redirected towards research projects due to the absence of clearly known pathogenic genes causing their respective phenotypes, while two other cases were rejected because their phenotypes were multifactorial with genetic predispositions identified through GWAS studies but without a known monogenic cause. One case was not accepted because a specific standard genetic test was judged more appropriate than HTS, and another case was rejected because it consisted of a prenatal diagnosis based on ultrasound findings without a specific hypothesis for a Mendelian disorder.

Once a case is accepted for HTS, the GCTF members discuss and define the most appropriate targeted gene panel. Since we use exome sequencing followed by bioinformatics selection of genes of interest, the clinical geneticists continuously reevaluate and update the gene lists. Before a gene panel is created or reevaluated, a detailed research of the literature is performed for recent review papers and available proposed gene panels (academic and commercial). Frequently used gene lists, such as the intellectual disability panel, are reviewed biannually or sooner at the request of the referring physician, while rarely used lists are reevaluated each time they are needed. As illustrated by the example, we initially used rather restrictive gene panels in order to minimize the incidental findings. In cases where no pathogenic variant was identified, we had the tendency to add a second or even a third bioinformatics analysis using additional genes. This multi-step approach was eventually deemed time consuming. Thus, we have recently decided to directly include all potentially causative genes in the panels.

After the pre-test GCTF decision, the patients or the legal representative are seen for a pre-HTS genetic consultation by one of the clinical geneticists (step 3). The patients are informed about the procedure and the possible results, including incidental findings in case of large panels. If they agree to be tested, we discuss and explain the specific HTS informed consent form, which we have developed in collaboration with the Swiss Society of Medical Genetics (ww.sgmg.ch) and with the input of the bioethicists. The patients can opt-in for the following categories of incidental findings, which are available only for the specific set of genes that will be analyzed:

1. Disorders for which no medical intervention (curative or preventive) is possible
2. Disorders for which medical intervention (curative or preventive) is possible
3. Carrier status for recessive disorders.

### HTS and bioinformatics analysis (steps 4, 5, and 6 of Fig. 2)

One important decision was the choice of the HTS strategy [14]. The different options included (i) WES and analysis of all genes implicated in Mendelian disorders; (ii) WES and bioinformatics targeted analysis of gene lists; (iii) targeted gene panels only, and (iv) whole genome sequencing (WGS) and bioinformatics targeted analysis of gene lists. Based on the previous experience from research projects [15, 16], we have chosen to perform whole exome sequencing (WES) followed by targeted analysis of specific sets of genes. Trio exome sequencing (patient and parents) was not considered due to limitations imposed by the Swiss medical insurance reimbursement regulations.

Exome capture is performed using the SureSelect Human ALL Exon technology (Agilent Technologies, Santa Clara, CA, USA), and the sequencing is realized in an Illumina HiSeq 2000 instrument (usually 100 bp paired-end, Illumina, San Diego, CA, USA). Read mapping, variant calling, and variant annotation are performed using a locally developed bioinformatics tool that surveys the sequential progress of the data from BWA [14] for mapping (hg19) and Samtools [15] and Pindel [16] for SNV and indel calling. Targeted bioinformatics analysis of the selected genes of interest is performed through locally developed pipelines, which select only the variants from the specified genes of interest, masking the rest of the data. We have validated the sequencing and variant detection quality by sequencing DNA samples from the individual for which sets of high-quality variants are publicly available (Platinum Genomes, Illumina®) and obtained 99.6 % concordance for the SNVs and 97.8 % for the indels. The bioinformaticians from the HTS section follow the updates of the databases (Table 1). New versions are implemented 1 to 3 months after they become publicly available. Software updates (Table 1) follow a more lengthy cycle and are annually reevaluated. Newer versions are implemented only when a significant improvement over previous results can be demonstrated.

**Table 1:** Databases and tools routinely used for variant annotation and classification. Additional databases and tools are used as deemed necessary

Population, disease-specific, and sequence databases	
Population databases	
Exome Aggregation Consortium	http://exac.broadinstitute.org/
1000 Genomes	http://browser.1000genomes.org
dbSNP	http://www.ncbi.nlm.nih.gov/snp
Disease databases	
ClinVar	http://www.ncbi.nlm.nih.gov/clinvar
OMIM	http://www.omim.org
Human Gene Mutation Database	http://www.hgmd.org
Leiden Open Variation Database	http://www.lovd.nl
Sequence databases	
NCBI Genome Source	http://www.ncbi.nlm.nih.gov/genome
RefSeqGene	http://www.ncbi.nlm.nih.gov/refseq/rsg
In-silico predictive algorithms	
Missense prediction	
SIFT	http://sift.jcvi.org
MutationTaster	http://www.mutationtaster.org
PolyPhen-2	http://genetics.bwh.harvard.edu/pph2
Splice site prediction	
GeneSplicer	http://www.cbcb.umd.edu/software/GeneSplicer/gene_spl.shtml
Human Splicing Finder	http://www.umd.be/HSF/
NetGene2	http://www.cbs.dtu.dk/services/NetGene2
NNSplice	http://www.fruitfly.org/seq_tools/splice.html
Conservation scores	
GERP	http://mendel.stanford.edu/sidowlab/downloads/gerp/
PhastCons	http://compgen.bscb.cornell.edu/phast/
PhyloP	http://compgen.bscb.cornell.edu/phast/

Potential pathogenicity of the variants (step 6) is evaluated by two senior scientists with extensive experience in the analysis of exome data. All analyses are performed independently and the results are then merged for the presentation and debate at the GCTF (step 7). Data pathogenicity estimation adheres to published guidelines [9] that has allowed the standardization of the process and has also guided a more structured approach towards the available databases (Table 1) that are now used in order to support or reject specific criteria. We are planning to set up a specific training program in order to increase the number of people involved and thus increase the capacity to perform exome analysis for the patients.

### Intermediate report and decisions on pathogenicity (step 7 of Fig. 2)

The intermediate report, produced for each patient, includes a technical and a variant interpretation section. The technical section documents all the HTS and bioinformatics analysis aspects including the specific filtering steps and quality metrics (e.g. genes, coverage, and number of identified variants). For the interpretation section we use the currently accepted 5 class variant classification system (8) and several tools for the classification (Table 1) [8, 9].

All class 3, 4, and 5 variants [9] are documented in the intermediate report with a summary of available literature and presented at the GCTF session (step 7). The classification performed by the analysis team is debated and occasionally the variants are reclassified after thorough evaluation of potential phenotype-genotype correlations. In cases where no clear pathogenic variant is found, it is discussed whether further analyses are warranted (e.g., MLPA for deletions/duplications, broader bioinformatics analysis that includes analysis of additional genes, Sanger sequencing of individual exons that are insufficiently covered) or if familial segregation analysis of a variant is justified. If necessary, we extend such segregation analysis up to second-degree relatives (cousins, nephews/nieces) but this depends mainly on disease status, demographic circumstances, and the interfamilial relationships.

### Verification, final report, and post-test considerations (steps 8 and 9 in Fig. 2)

All identified variants (100 %) that are disclosed in the final report are currently confirmed by Sanger sequencing and the content of the final reports are discussed during the GCTF sessions (step 8). All class 4 and 5 variants identified in genes compatible with the phenotype are reported. The disclosure of class 4 and 5 variants considered as incidental findings is done according to the patient’s pre-test decision. Class 3 variants are only disclosed if they are found in a gene causing a phenotype which is compatible with the clinical presentation of the patient. In these cases, a remark that the variants should be reevaluated in 1–2 years according to the evolving knowledge is added.

Once the final report has been validated by the GCTF, the report is signed by the senior molecular biologist, allowing the clinical geneticists to subsequently arrange genetic counseling for communication of the results (step 9).

### Example illustrating the operating procedure of the GCTF

Dizygotic 12-month-old male twins were addressed for genetic counseling because of seizures since the age of 4 months associated with severe developmental delay. Family history was unremarkable. An array-CGH (resolution 180 KB) was performed and identified a 417 kb paternally inherited duplication at 6p12.2 that was considered non-pathogenic. Extensive paraclinical workup and brain imaging did not reveal the cause. The clinical geneticist in charge presented the situation at the weekly GCTF meeting. Given the lack of diagnosis and the severe presentation, we decided to perform WES with targeted bioinformatics analysis of 120 selected epilepsy genes. This initial analysis did not reveal any potential pathogenic variants. It was then decided to extend the analysis by including all known syndromic and non-syndromic epilepsy genes. The second panel consisted of 395 genes and revealed a novel missense variant NM_020473.3:c.481G>A: p.(Glu161Lys) in the gene *PIGA* on chromosome Xp22.2 (MIM 311770). This variant concerns a very well-conserved nucleotide (GERP: 5.89) and was predicted to be pathogenic by all three bioinformatics tools (SIFT: 0, PolyPhen/HumVar:0.931, Mutation Taster:0.999). Pathogenic mutations in *PIGA* cause paroxysmal nocturnal hemoglobinuria (MIM 300818) and multiple congenital anomalies-hypotonia-seizures syndrome 2 (MCAHS2, MIM 300868). The latter phenotypic description was concordant with the children’s clinical presentation. The variant was transmitted from their unaffected mother. No additional family members were available for clinical testing. Based on the aforementioned evidence, the variant was reported as pathogenic. Our approach allowed us to expand the bioinformatics analysis to additional genes without the need for resequencing; however, the turnaround time was prolonged as we did not immediately include all potentially causative genes. Based on this experience, and on similar other situations, we decided to change our procedure and to analyze directly the largest possible gene panel.

### Reimbursement of HTS for Mendelian disorders

Another aim of the GCTF was to initiate together with the Swiss Society of Medical Genetics the administrative process with the Swiss Federal Office of Public Health (SFOPH) in order to integrate HTS as a reimbursable genetic test in the Swiss health care system. Genetic tests in Switzerland are reimbursed according to a positive “list of analyses” (LA) [17], which specifies each disorder and testing method covered by the compulsory, albeit private, medical insurance scheme. The LA includes an additional nonspecific entry for orphan diseases, applicable to rare Mendelian disorders, not otherwise registered in the LA. In January 2015, after 30 months of continuous negotiations with the Swiss Federal Office of Public Health, HTS was officially introduced in the LA as a reimbursable genetic test for Mendelian disorders [17]. In addition, the Swiss Society of Medical Genetics (SSGM) [18] developed a document of “good practice” for the use of HTS in clinical setting [19], which was required by the SFOPH, covers pre- and post-HTS genetic counseling issues, an informed consent form adapted to HTS genetic testing, laboratory requirements and specifications, regulations for secure data storage and quality control, and disclosure of secondary findings and variants of unknown clinical significance, as well as recommendations for the reporting of results.

The costs of HTS are based on the sum of three distinct entries within the LA: laboratory costs of high-throughput sequencing, bioinformatics analysis, and additional confirmatory laboratory analyses such as Sanger sequencing and/or MLPA. More specifically:

1. High-throughput sequencing has a fixed price of 2300 CHF, irrespective of the sequencing technology used (WES or targeted panel approach).
2. Bioinformatics analysis costs vary according to the number of genes analyzed: 600 CHF for 1-10 genes, 1000 CHF for 11-100 genes and 1500 CHF for more than 100 genes.
3. Confirmation of variants using Sanger sequencing (215 CHF per variant): a maximum of two Sanger confirmations for 1–10 genes, four for 11–100 genes, and six for more than 100 genes. In all cases, a maximum of four multiplex ligation-dependent amplification (MLPA) analyses can also be added to the total cost (350 CHF per MPLA).

This modular setting enables flexibility for the diagnostic laboratories and allows each step to be performed and charged separately. In particular, it allows performing additional bioinformatics analyses without resequencing, which is arguably cost-effective.

Additional requirements have been set forth by the SFOPH for the reimbursement of HTS: diagnostic laboratories performing HTS must participate in quality assessment schemes (EQAs), according to the Swiss law [20], and become accredited for HTS by the Swiss Accreditation Service (SAS), before December 31, 2017. Furthermore, all the steps of HTS need to be performed within Switzerland. However, it is not necessary that they are all performed within the same institution. Finally, because of the complexity of pre- and post-HTS counseling, only board-certified medical geneticists [21] are allowed to prescribe HTS tests of more than 10 genes. Physicians from all other medical specialties can only prescribe HTS for less than 10 genes. In addition, the requirements for expert genetic counseling are well specified in the existing law for genetic analyses [22]. It is planned to regularly update and reevaluate the requirements from the SFOPH as well as the “good practice” document according to the new developments and international recommendations in the field.

### Experience to date

Until now we have designed 51 different gene lists containing 2 to 1038 genes. On average, 160 (SD = 18) million reads are produced per sample. After removal of duplicate reads, 132 (SD = 22) million reads remain, 78 (SD = 8.5) million of which are on target (target = total coding sequence as defined by RefSeq). These reads represent an average coverage of the coding portion of the RefSeq genes of at least 20× for 94.73 % (±1.18 SD) and of at least 30× for 92.08 % (±1.66 SD). On average, 21,565 (±1,125 SD) variants (SNVs and small indels) are detected per individual.

So far, we enrolled 240 patients with our HTS approach. Thirty-two percent (77/240) displayed developmental delay with or without other anomalies; the remaining 68 % (163/240) presented with various heterogeneous Mendelian disorders such as short rib polydactyly syndrome, juvenile Parkinson disease, connective tissue disorders, Cornelia de Lange syndrome, microcephalic primordial dwarfism (MPD), Kallmann syndrome, arthrogryposis, Gitelman syndrome, various inherited cardiac diseases, Charcot-Marie-Tooth disease, Kabuki syndrome, hereditary spastic paraparesis, or likely monogenic epilepsy. We completed the final report for 139 of these patients (47 with developmental delay and 92 with other Mendelian disorders).

**Fig. 3:**
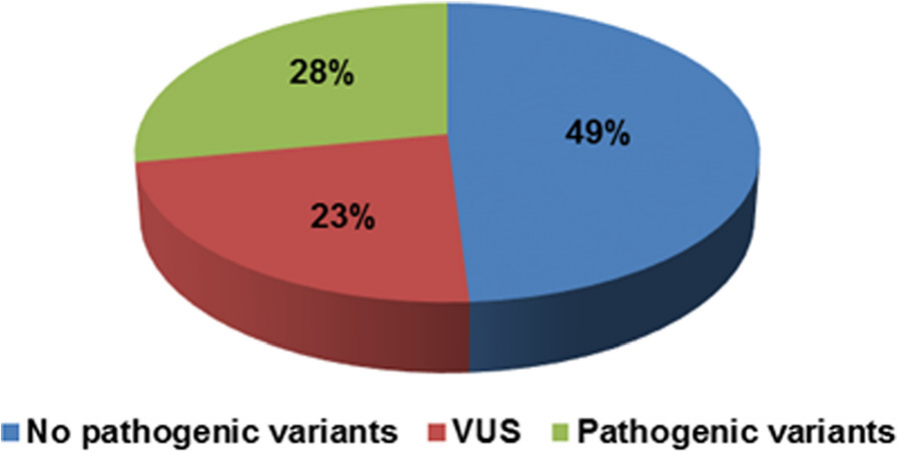
Results of targeted gene analysis in 47 patients with developmental delay. Pathogenic variants were identified in 28 %, VUS in 23 %, and no pathogenic variants were found in 49 % of the patients, respectively

**Fig. 4:**
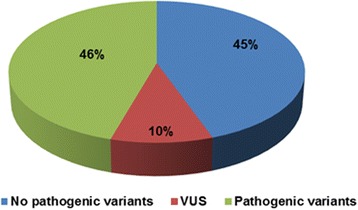
Results of targeted gene analysis in 92 patients with various Mendelian diseases. Pathogenic variants were found in 46 %, VUS in 10 %, and no pathogenic variants were found in 45 % of the patients, respectively

Pathogenic variants (class 4 or 5) were detected in 28 % (13/47) of the patients with developmental delay (Fig. 3) and in 46 % (42/92) of the patients with other Mendelian phenotypes (Fig. 4), which gives an average detection rate of class 4 or 5 variants of 40 % (55/139) (Table 2). Variants of unknown clinical significance (VUS, class 3) were found in 23 % (11/47) of the patients with developmental delay (Fig. 3) and in 10 % (9/92) of the patients with other Mendelian phenotypes (Fig. 4). So far, we reported 8 out of 20 identified VUS in the final report.

**Table 2:** Causative variants identified in the resolved cases

	Phenotype	Gene panel	Identified pathogenic variant(s)
1	Short rib polydactyly	Short rib polydactyly panel (10 genes)	NM_001377.2(DYNC2H1_v001):c.1953G>A:p.(=)
			NM_001377.2(DYNC2H1_v001):c.4625 C>T:p.(Ala1542Val)
2	Severe ID	ID (536 genes)	NM_000489.4(ATRX_v001):c.6122G>A:p.(Ser2041Asn)
3	Intellectual disability, microcephaly	ID (536 genes)	NM_004380.2(CREBBP_v001):c.4665A>C:p.(Glu1555Asp)
4	Cornelia de Lange syndrome	Cornelia de Lange panel (5 genes)	NM_015384.4(NIPBL_v001):c.5483G>A:p.(Arg1828Gln)
5	Intellectual disability	ID (536 genes)	NM_004187.3(KDM5C_v001):c.769_770del :p.(Leu257Alafs*5)
6	Glomerulopathy	Glomerulopathy and Alport panel (61 genes)	NM_000495.4(COL4A5_v001):c.2288G>A:p.(Gly763Glu)
7	Intellectual disability, psychotic symptoms	ID (536 genes)	NM_033517.1(SHANK3_v001):c.3637dup:p.(His1213Profs*83)
8	Microcephalic primordial dwarfism	MPD panel (18 genes)	NM_002312.3(LIG4_v001):c.2321T>C:p.(Leu774Pro)
			NM_002312.3(LIG4_v001):c.2440C>T c.2440 C>T p.(Arg814*)
9	Kallmann syndrome	Kallmann panel (21 genes)	NM_015850.3(FGFR1_v001):c.1444del:p.(Leu482Trpfs*25)
10	Dyskinesia, dystonia, myoclonia	Dystonia panel (8 genes)	NM_003919.2(SGCE_v001):c.783dup :p.(Phe262Ilefs*8)
11	Cardiac arrest	Cardiomyopathy panel (66 genes)	NM_001035.2(RYR2_v001):c.14711G>A:p.(Gly4904Asp)
12	Periodic fever syndrome	Periodic fever panel (4 genes)	NM_004895.4(NLRP3_v001):c.1049C>T:p.(Thr350Met)
13	Intellectual disability, microcephaly, strabismus	ID (536 genes)	NM_021140.3(KDM6A_v001):c.3598C>T :p.(Leu1200Phe)
14	Hereditary spastic paraplegia	Hereditary spastic paraplegia panel (45 genes)	NM_014846.3(KIAA0196_v001):c.1857G>C:p.(Leu619Phe)
15	Epileptic encephalopathy	Epilepsy panel (395 genes)	NM_020473.3(PIGA_v001):c.481G>A:p.(Glu161Lys)
16	Gitelman syndrome	Gitelman syndrome panel (2 genes)	NM_000339.2(SLC12A3_v001):c.1924C>G:p.(Arg642Gly)
17	Autism, Intellectual disability, trigonocephaly	ID (536 genes)	NM_001111125.2(IQSEC2_v001):c.2477T>C:p.(Met826Thr)
18	Aortic dissection	Aneurysm panel (20 genes)	NM_000138.4(FBN1_v001):c.6616G>A:p.(Asp2206Asn)
19	Epileptic encephalopathy	Epileptic encephalopathy (141 genes)	NM_004518.4(KCNQ2_v001):c.821C>T :p.(Thr274Met)
20	Kabuki syndrome	Kabuki panel (2 genes)	NM_003482.3(KMT2D_v001):c.12661C>T:p.(Gln4221*)
21	Hereditary Spastic paraparesis	Spastic paraparesis panel (11 genes)	NM_199436.1(SPAST_v001):c.1015C>T :p. (Leu339Phe)
22	Ohdo syndrome	KAT6B gene	NM_001256468.1(KAT6B_v001):c.4652_4661dup:p.(Gln1554Hisfs*41)
23	Neurofibramotosis type 1	NF panel (2 genes)	NM_000267.3 (NF1_v001):c1381C>T: p.(Arg461*)
24	Inclusion body myositis	Inclusion body myosotis panel (10 genes)	NM_001927.3 (DES_v001):c.1155G>T:p.(Asp399Tyr)
25	Noonan syndrome	Noonan and rasopathy syndrome (12 genes)	NM_002834.3 (PTPN11_v001):c.797G>C:p.(Glu139Asp)
26	Periodic fever	Personalized periodic fever panel (207 genes)	NM_000243.2 (MEFV_v001):c.2084A>G:p.(Lys695Arg)
27	Charcot Marie Tooth type 2	CMT2 panel (23 genes)	NM_001005373.3 (LRSAM1_v001):c.2069T>C:p.(Cys690Arg)
28	Hypoglycemia on congenital hyperinsulinemia	Congenital hyperinsulinemia panel (10 genes)	NM_000525.3 (KCNJ11_v001):c.400T>C:p.(Leu147Pro)
			NM_000525.3 (KCNJ11_v001):c.154C>T:p.(Gln52*)
29	Cardiomyopathy	Cardiomyopathy panel (66 genes)	NM_001018008.1 (TPM1_v001):c.304G>A:p.(Glu102Lys)
30	Intellectual disability, epilepsy	Intellectual disability panel (537 genes)	NM_000834.3 (GRIN2B_v001):c.1598G>A:p.(Gly533Asp)
31	X-linked intellectual disability	Intellectual disability panel (990 genes)	NM_003916.4 (AP1S2_v001):c.1-3C>A
32	Lissencephaly	Lissencephaly panel (12 genes)	NM_000403.3 (PAFAH1B1_v001):c.162dupA:p.(Trp55Metfs*6)
33	Vascular leukoencephalopathy	Vascular leukoencephalopathy panel (7 genes)	NM_002775.4 (HTRA1_v001):c.854C>T:p.(Pro285Leu)
34	Cardiomyopathy	Cardiomyopathy panel (66 genes)	NM_000256.3 (MYBPC3_v001):c.3324-3325del:p.(Lys1108Asnfs*41)
35	Cardiomyopathy	Cardiomyopathy panel (66 genes)	NM_000256.3 (MYBPC3_v001):c.3697C>T:p.(Gln1233*)
36	Cardiomyopathy and connective tissue disorder	Cardiomyopathy and connective tissue disorder panel (166 genes)	NM_0004415.2 (DSP_v001):c.4003C>T:p.(Gln1335*)
37	Intellectual disability	Intellectual disability panel (990 genes)	NM_002834.3 (PTPN11_v001):c.794G>A:p.(Arg265Gln)
38	Cystinuria	Cystinuria panel (2 genes)	NM_001243036 (SLC7A9_v001):c.1225-4678_1324del
39	Noonan syndrome	Noonan panel (12 genes)	NM_002834.3 (PTPN11_v001):c.923A>G:p.(Asn308Ser)
40	Intellectual disability, microcephaly	Personalized panel (2 genes: *DYRK1A* and *DDX3X*)	NM_00139.3 (DYRK1A_v001):c.1491delC:p.(Ala498Profs*94)
41	Neonatal encephalopathy	Encephalopathy panel (225 genes)	NM_001909.4 (CTSD_v001):c.686_688del:p.(Phe229del)
42	Intellectual disability, cryptorchidism	Intellectual disability panel (990 genes)	NM_001243234.1 (TCF4_v001):c.656dupT:p.(Leu219Phefs*9)
43	Intellectual disability, obesity	Intellectual disability panel (990 genes)	NM_032531.3 (KIRREL3_v001):c.2019G>A:p.(Met673Ile)
44	Epilepsy, vertigo, episodic ataxia	Epilepsy (396 genes)	NM_0010540143.1 (SCN2A_v001):c.2960G>T:p.(Ser987Ile)
45	Intellectual disability	Intellectual disability panel (990 genes)	NM_015559.2 (SETBP1_v001):c.2016-2017insT:p.(Lys673*)
46	Kabuki syndrome	Kabuki panel (2 genes)	NM_003482.3 (KMT2D_v001):c.2994delT:p(Met999*)
47	Long QT syndrome	Arythmia panel (47 genes)	NM_000238.3 (KCNH2_v001):c.1786C>G:p(Pro596Ala)
48	Rubinstein-Taybi syndrome	Rubinstein-Taybi syndrome panel (2 genes).	NM_004380.2 (CREBBP_v001). Variant found by MLPA
49	Aneurysm and dyslipidemia	Aneurysm and dyslipidemia panel (50 genes)	NM_000041.3 (APOE_v001):c.461G>T:p.(Arg154Leu)
50	Marfan syndrome	Marfan syndrome panel (8 genes)	NM_000138.4 (FBN1_v001):c.7339G>A:p.(Glu2447Lys)
51	Ehlers-Danlos syndrome	Ehlers-Danlos panel (4 genes)	NM_000093.4 (COL5A1_v001):c.2203dupC:p.(Gln735Profs*25)
52	Epileptic encephalopathy and intellectual disability	Intellectual disability and epilepsy panel (1038 genes)	NM_001127648.1 (GABRA1_v001):c.641G>A:p.(Arg214His)
53	Intellectual and communication disability	Whole exome	NM_001197104.1 (MLL/KMT2A_v001):c.2633G>A:p.(Arg878Gln)
54	Catecholaminergic polymorphic ventricular tachycardia, arrhythmia	Cardiomyopathy panel (66 genes)	NM_001018008.1 (TPM1_v001):c.304G>A:p.(Glu102Lys)
55	Dilated non compaction cardiomyopathy	Arythmia and cardiomyopathy panel (97 genes)	NM_003319.4 (TTN_v001):c.49905dup:p.(Pro16636Thrfs*9)

Sanger sequencing of not well-covered exons in genes that were considered to be highly compatible with the phenotype was performed in two cases (2/139, 1.4 %) and in one of them the causative variant was identified. In six other cases (6/139, 4.3 %), bioinformatics analysis of further added genes to the originally determined gene panel resulted in the identification of the causative variants.

### Lessons learned

HTS has provided exciting new diagnostic opportunities in the investigation of genetically heterogeneous Mendelian disorders and has expanded the capacity to test simultaneously a large number of candidate genes in a timely fashion and for a reasonable cost. The advantages of our approach include the following: (i) the use of one common diagnostic test for all patients suffering from Mendelian disorders with known pathogenic genes, (ii) it is amenable to a customized and flexible bioinformatics analysis, (iii) it allows us to invite all patients with a negative result to contact us again within an interval of 1–2 years, in order to reevaluate the results and possibly expand the analysis according to new discoveries without the need to re-sequence, (iv) it minimizes the probability of incidental findings not related to the patient’s disease.

However, WES followed by targeted bioinformatics also has drawbacks. Firstly, from the sequencing depth and coverage point of view, the capture procedure of the whole exome is less efficient when compared to well-established targeted gene panel enrichment. Nevertheless, with the use of the most recently marketed whole exome capture reagents, this is gradually becoming less of an issue [23], although there is a need to continuously monitor the coverage. This drawback may be partially resolved by filling up the sequencing gaps using traditional methods such as targeted Sanger sequencing of poorly covered exons. This complementary procedure was recently introduced in the proposed draft of the Eurogentest guidelines for next-generation sequencing [24].

The second difficulty concerns the selection of genes of interest, which is not yet standardized, and thus remains idiosyncratic and to some extent subjective. Although our approach allows a very flexible and liberal selection of genes, a human omission or a change in the gene name increases the risk of false negative results. Furthermore, the possibility exists to include, particularly in large gene lists such as the one for intellectual disability, some actionable genes with broad phenotypic spectrum of manifestations or not directly linked to the investigated phenotype. Our experience has shown that it is crucial to regularly reevaluate the gene lists, ideally by two independent individuals using criteria adapted for diagnostic testing, such as the level of evidence for pathogenicity and its correlation with a known phenotype. Yet, despite rigorous monitoring of the literature, the risk of wrongful inclusion or exclusion of genes remains. We strongly encourage the establishment of international norms and criteria for selecting gene panels, in order to render the diagnostic possibilities universal. For example, achievements such as the release of the Eurogentest guidelines are a welcome development [25].

We do not routinely perform trio sequencing (father, mother, affected offspring) despite the fact that the trio approach seems to have a slightly higher diagnostic yield [26]. The main reasons for this decision are financial, as the health insurances only reimburse the HTS costs of the proband but not that of the parents.

The most demanding challenge in clinical HTS arguably remains the issue of variant interpretation. Since the number of variants identified is roughly proportional to the number of analyzed genes, the task of variant interpretation rises accordingly. In our group, each variant’s pathogenicity is first assessed by the analysis team according to international guidelines [9] and then presented, discussed, and evaluated for concordance with the phenotype of the patient during the GCTF sessions. The input of expert physicians familiar with the patient’s phenotype is invaluable as well as the interpretation workup and knowhow of the laboratory team. The emerging databases for the pathogenicity of variants are also extremely important, and sharing of the interpretation of variants among diagnostic centers is crucial. Clinical knowledge and experience help to (mostly) exclude variants as non-relevant to the phenotype or to consider them as likely pathogenic [9, 10]. This complementary exchange is in our opinion indispensable for adequate variant interpretation, especially in cases of large gene lists with several potentially pathogenic variants identified. Accordingly, in a few cases, the consensus of GCTF was to consider the identified variant as likely pathogenic (class 4), despite VUS classification (class 3) by the analysis team according to the guidelines [9].

In order to further facilitate and improve variant interpretation, the need for international sharing of variants and phenotypes is of paramount importance and cannot be overemphasized. We have put in place a semi-automatic submission of variants of classes 3/4/5 in ClinVar. Furthermore, false positive “pathogenic” variants that have been misclassified in the past need to be updated in relevant databases, so that false diagnoses will not be perpetuated.

In parallel to the technical advances, ethical aspects have to be constantly considered at the present stage of HTS genetic testing. One important issue concerns the pre- and post-HTS genetic counseling challenges, including informed consent. The expert participation of ethicists within the GCTF was of considerable value for the development of a specific informed consent form for HTS application in accordance with the Swiss law on genetic testing [22] and also for the continuous reevaluation of our procedures. The informed consent form respects the rights to know and not to know, especially concerning secondary findings in actionable genes and carrier status for recessive disorders. Our experience to date has shown that the majority of patients and parents want to know about treatable diseases or diseases for which effective preventive measures exist, but decide not to know about non-treatable disorders and carrier status. Additionally, almost all have agreed that their DNA and sequencing data could be stored for prospective future research projects. All these aspects need to be systematically studied on large cohorts in order to provide statistically meaningful conclusions.

A further key effort of the GCTF together with the Swiss Society of Medical Genetics was regarding the reimbursement of HTS as a diagnostic test by the insurance companies. It necessitated 2.5 years of continuous negotiations with the SFOPH until the proposal for formal reimbursement was accepted by the federal health authorities. To our knowledge, Switzerland is the first European country for which a specific formal policy for reimbursing of HTS for Mendelian disorders has been introduced. In a few European countries, reimbursement is achieved through general genetic testing policies; while in most other countries, HTS is still being funded by research projects or by non-reimbursable payments from the consumers. We hope that reimbursement policies will be developed in other countries in order to achieve a widespread acceptance and use of HTS for the diagnosis of genetic disorders.

In conclusion, the multidisciplinary GCTF has allowed us to implement a number of local procedures and criteria necessary to ensure high standard clinical services within the new field of diagnostic HTS, as well as to achieve in collaboration with the Swiss Society of Medical Genetics the formal federal decision for HTS reimbursement for monogenic disorders.
